# From Marine Venoms to Drugs: Efficiently Supported by a Combination of Transcriptomics and Proteomics

**DOI:** 10.3390/md15040103

**Published:** 2017-03-30

**Authors:** Bing Xie, Yu Huang, Kate Baumann, Bryan Grieg Fry, Qiong Shi

**Affiliations:** 1 Venomics Research Group, BGI-Shenzhen, Shenzhen 518083, China; xiebing@genomics.cn; 2 Shenzhen Key Lab of Marine Genomics, Guangdong Provincial Key Lab of Molecular Breeding in Marine Economic Animals, BGI, Shenzhen 518083, China; huangyu@genomics.cn; 3 Venom Evolution Lab, School of Biological Sciences, University of Queensland, St. Lucia 4072, Australia; kate.baumann@uqconnect.edu.au; 4 BGI Shenzhen Academy of Marine Sciences, BGI Fisheries, BGI, Shenzhen 518083, China

**Keywords:** marine toxins, database, transcriptome, proteome, venomics

## Abstract

The potential of marine natural products to become new drugs is vast; however, research is still in its infancy. The chemical and biological diversity of marine toxins is immeasurable and as such an extraordinary resource for the discovery of new drugs. With the rapid development of next-generation sequencing (NGS) and liquid chromatography–tandem mass spectrometry (LC-MS/MS), it has been much easier and faster to identify more toxins and predict their functions with bioinformatics pipelines, which pave the way for novel drug developments. Here we provide an overview of related bioinformatics pipelines that have been supported by a combination of transcriptomics and proteomics for identification and function prediction of novel marine toxins.

## 1. Introduction

A variety of biological activities have been identified in venoms, including neurobiological, enzymatic, cytotoxic, antibacterial, agglutination, hemolytic, anti-thrombus, coagulation, immunoregulatory, enzyme immune, and antiviral activities [1,2,3,4,5]. A typical example is that each subtype of Na^+^, K^+^, Ca^2+^ or Cl^−^ ion channels in almost all animals has its interactional venom peptides or proteins from different venomous species [6].

**Figure 1:**
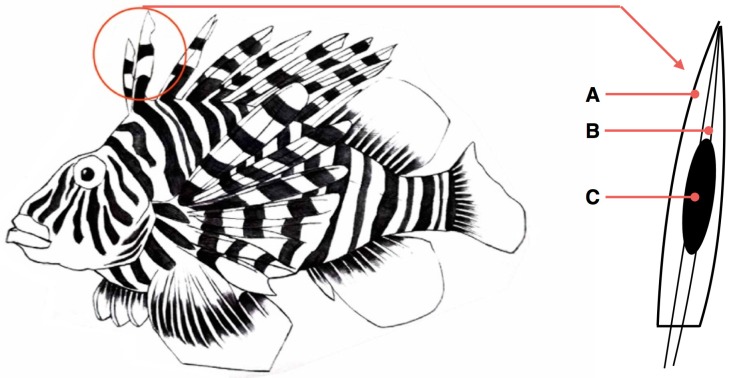
Morphology of venom glands in a scorpionfish. For venom fish, venom glands are usually located in their pectoral and dorsal fins. As shown in the right enlarged image, venom spines are typically composed of spine (**A**), connective tissue (**B**) and venom gland (**C**).

Marine venoms have been largely ignored as a source for potential pharmaceuticals, despite research suggesting that there are more marine venomous species than all other venomous terrestrial animals combined [7]. Little is known about the composition of marine venoms and, consequently, these venoms present a unique source of novel drugs and pharmacological tools. Bioassay-guided fractionation has been traditionally used for marine venom analysis [8]. However, this approach is considered time-consuming and requires large amounts of crude venoms, which are not always available. The extraction of venoms from the venom gland tissues is also troublesome as marine venoms have been shown to be highly labile and sensitive to heat, changes in pH, lyophilization, storage or repeated freeze-thaw cycles [9]. Marine venom samples are typically mucus-rich, causing immense difficulty during proteomic methodologies. The collection of fish venoms has proven to be the most difficult issue as the venom glands are typically deeply embedded in the skin or muscle of the venom apparatus, and it is impractical to remove the venom gland without interfering with peripheral tissues (Figure 1).

Multi-omics studies using next-generation sequencing (NGS) and liquid chromatography-tandem mass spectrometry (LC-MS/MS) technologies advanced considerably, leading to more sensitive and efficient research of venoms [10,11]. These techniques have been proven to be successful in several fields, such as neuroendocrine research and drug discovery [12]. Further, utilization of de novo assembling algorithms for deep sequencing has been widely applied in large-scale genomic and transcriptomic sequencing projects, with accurate assembly of fragment data into full-length transcripts, in particular in the absence of a reference genome sequence [13]. 

In this review, we access the current state of knowledge regarding marine venoms, in particular how toxin databases can be correctly utilized in order to accurately predict function of marine toxins.

## 2. Toxin Database

There are two kinds of toxin databases, generalist and toxin-centered. In generalist databases such as Genbank (a collection of all publicly available sequences), it is difficult to extract the toxin sequences or their structure data due to a lack of annotations as toxins or the redundancy of similar sequences [11,14]. Large amounts of toxin information have been submitted along with publications; as a consequence, these data show up in the peer-reviewed literature rather than in generalist databases. In contrast, most sequences in toxin-centered databases have been well annotated and peer-reviewed [8]. The Tox-prot Program [15], the Animal Toxin Database (ATDB) [16], ConoServer [17], ArachnoServer [18,19], and ISOB (Indigenous Snake species Of Bangladesh, http://www.snakebd.com/) provide expert annotations on sequences and 3D structures of general venomous animals [16,19,20,21,22]. Sequences from these toxin-specific databases can be easily traced back to the original peer-reviewed papers or found in the generalist databases. Databases such as Conoserver and ArchnoServer are good at addressing the problem of nomenclature of newly identified toxins [23,24].

A complete and well-annotated sequence provides the ultimate resource for venomics approaches; however, this relies on the accuracy of toxin sequences from a given database in order to predict if the sequence is a toxin or not. Toxin sequences share many similarities in their sequences, further increasing the difficulty in accurately annotating the sequences.

After a brief survey of related publications and these above-mentioned public databases, we found that unlike the databases for venom terrestrial animals (i.e., scorpions, spiders and snakes), there is no such unique toxin database or dataset for marine venomous species except cone snails (ConoServer Database), which is a major drawback in the research of marine venoms [25]. Despite general databases, such as NCBI-RefSeq, NCBI-nucleotide, UniProtKB/Swiss-Prot and TrEMBL, being available, toxin and non-toxin sequences are combined, making it difficult to extract the required sequences. These difficulties make alignment work redundant and time-consuming. However, a comprehensive in-house database has been constructed [7] to cover currently annotated toxin sequences of reported venomous species (Table 1).

Our in-house toxin database is a comprehensive dataset of all public toxin sequences, which enables the discovery and annotation of toxin genes. There were 4455 toxin sequences identified from venomous marine animals, 87% of which were from cone snails (Table 1). Considering the remarkable research done on cone snails, it is not surprising that the majority of sequences came from these species. However, it highlights the insignificant number of sequences from other venomous marine species that have been discovered. There are still many obstacles to overcome this scarcity of sequences on venomous marine species. For example, traditional annotation strategies using Blast2Go and other programs in order to annotate assembled sequences are unsuccessful in many cases due to rare homologs of toxins present in public databases. This issue hence complicates creation of bioinformatics pipelines.

## 3. Venom-Gland Transcriptomics

Due to the dramatic decline of the cost for NGS sequencing, there are a large number of transcriptomes available for snakes, spiders, scorpions and many other terrestrial venomous animals. Except for the identification of novel biological active toxins, evolution/diversities of toxin families and discovery for drug precursors are also included in the hottest research fields [26,27,28].

Related transcriptomics analysis can identify all the toxin genes transcribed under certain biological circumstances or certain ecological environments. Transcriptomes can also provide insights into the mechanisms and the diversity of toxins, venom synthesis and secretion, and the biological functions of venoms. Meanwhile, comparative transcriptome analysis allows parallel examination of the dynamic expression of all genes in a holistic manner. This contributes to understanding the unique biological functions of the venom glands. A recent study undertaken on the venom glands of fish shows that the glands most likely originate from the skin and the secretions from the skin are speculated to play an important part in the skin recovery and immunity [29].

**Figure 2:**
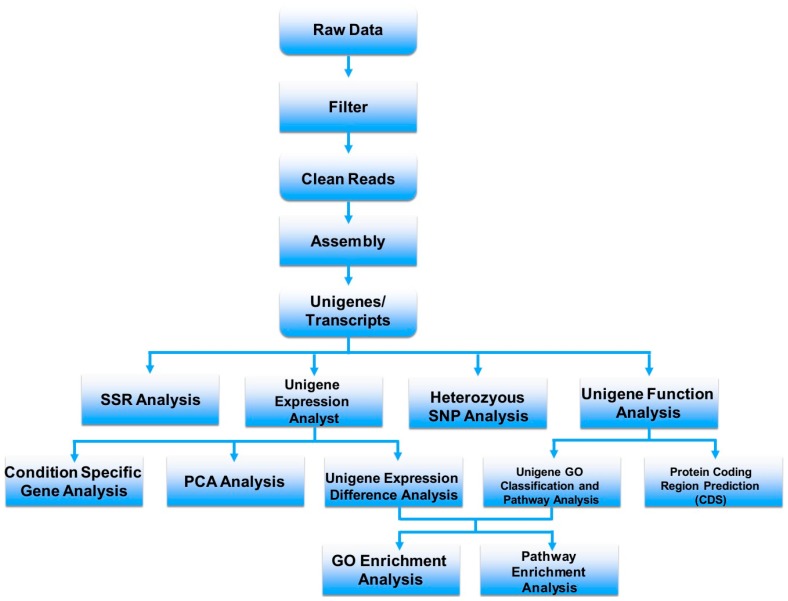
A standard procedure for transcriptome analysis at BGI.

The method for the transcriptome analysis of venom glands is summarized in Figure 2, which is modified from a standard procedure at BGI [30]. In brief, raw reads are firstly trimmed and subsequently eliminated to remove redundant and low-quality reads, before assembling into contigs. The functions of contig genes are further predicted by homologies extracted from public databases such as NCBI/Nr [31] and/or UniProtKB [32]; toxin precursors are then identified among the contigs for further analysis and classification. Usually, these reads and contigs are required to be stored in one of several public generalist repositories, such as NCBI SRA [31].

Illumina sequencing platforms are the most widely used due to their high outputs and long reads [7]. Transcriptomic sequencing is valuable for the venomous species whose de novo assembled whole genome sequences are absent (i.e., without a reference genome). However, assembling of these transcriptome reads is still considered challenging and should be treated with caution, since only a few venomous genomes and/or transcriptomes are available. Currently, most related sequences are from three snake species (Bermese python, king cobra and five-pacer viper) [33,34,35], two cone snails (*Conus bullatus* and *C. consors*) [36,37], one scorpion [38], one spider [39], a honeybee [40] and parasitic wasps [41]. 

For the majority of venomous marine species, the parameters for assembly software should be carefully scrutinized. Generally speaking, the assembling strategy will vary for different species, since the guanine-cytosine (GC) content, N50, and the mean length for evaluating the quality of assembly will always be various in different species. Different assembly softwares and parameters are often comparable and their performances are often assessed on the basis of annotation results. While looking into the results of annotations, we always find that toxin precursors can be aligned to several highly divergent superfamilies, which might be confusing for our subsequent analysis. We previously observed that fish toxins belonging to any novel gene superfamily are difficult to identify using sequence similarities due to the remote phylogenetic relationships between our examined fish and those species in the public databases. 

Reported studies have shown that hundreds of thousands of toxins may originate from only a few primitive genes [42,43]. Scholars have reached a consensus that in the long evolutionary history of venomous species, only a few primitive genes have been recruited. These genes originally functioned as non-venoms (such as hormone, proteinase inhibitor, nerve growth factor, lectin and so on) before gradually encoding as toxin peptides or proteins under evolutionary pressure [27,44,45]. Based on these theories and as a solution for gene annotation, profile-based alignments are more credible since their arithmetic has been based on the position-scoring matrices of conservative sites and further applied on a few studies for analyzing venom gland transcriptomes [7,46,47,48]. Profile-hidden Markov models (pHMMS) have been recently used to identify toxin transcripts in several cone snails and fish transcriptomes [7,46,47,49,50,51]. 

## 4. Venom-Gland Proteomics

Similar to those approaches for terrestrial species [25,52], proteomics approaches applied in marine venomous animals include chromatography, electrophoresis, enzymatic digestions, Edman degradation, and mass spectrometry (MS) [53,54].

Traditional proteomics relies largely on the use of automated Edman degradation and amino acid composition analysis, followed by the confirmation of molecular weights. This approach enables confident assignment of peptide sequences; however, it suffers from both low throughput and a large amount of sample demand. However, there are typically hundreds of different peptides in the venom of a specific venomous species [55], and therefore sequencing by Edman degradation will be prohibitively expensive for the large number of peptides. Fortunately, in recent years, the development of highly sensitive and high-resolution MS instruments to provide novel fragmentation techniques has established a new solution to these issues. Most toxins are very short in sequences and hence can be sequenced at a lower cost using tandem MS (MS/MS) [3,52,56], but Edman degradation still can be useful as a complement to MS. For example, the latter can help to identify the isobaric amino acids isoleucine/leucine and for N-terminal sequencing [57].

Until recently, the studies of toxic peptides from marine venomous animals have been mostly limited by the isolation and biochemical characterization of toxins of medical importance. Little or no attention was paid to the related genes, cellular machinery, and other important processes involved in assembly of the final products expressed in the venoms. Marine animal venoms were generally screened in medium- to high-throughput assays against targets of therapeutic interest, and then “hit venoms” were chromatographically fractionated and the individual fractions were re-screened in order to isolate peptides responsible for bioactivity. In some cases, incomplete sequence information acquired via MS/MS and/or Edman degradation has been used for designing primers to amplify transcripts encoding the toxin of interest from a venom gland cDNA library. This method has the advantage of providing useful information about the signal and pro-peptide regions of the toxin precursors as well as the sequences of transcripts encoding paralogs (and even orthologs in related species) [58]. 

Most of the known toxin sequences were predicted from RNA sequences with six frame-translating or open reading frame (ORF)-finding tools. Consequently, the majority of toxins cataloged in public databases do not have any experimental support (at protein or activity levels) for their production in venoms. For instance, there are 1873 mature toxins recorded in Conoserver, while only 379 toxins have experimental evidence. However, supports for mature toxin sequences are increasing rapidly with evidence from modern proteomic experiments.

Throughout the course of evolution, venom peptides and proteins from both vertebrates and invertebrates have been optimized to target specific receptors with high affinity and often exquisite selectivity, making them excellent pharmacological tools and drug leads [59,60,61]. The number of venom-derived peptides in preclinical or clinical trials has been increasing significantly in the past two decades [59].

## 5. Combination of Transcriptomics and Proteomics

Over the past few years, there has been a rapid development in transcriptomics and proteomics research for toxins on the basis of a combination of NGS and MS. The multi-omics analysis on a venom gland (i) can reveal the toxin genes under certain biological conditions or ecological environments; (ii) can provide useful information for the scale and mechanism of the variety of toxins; (iii) and can provide solutions to those biological questions concerning toxin functions, the process of toxin synthesis and the secretion of toxin peptides. Meanwhile, there is a special significance for the research targets that have never been studied before with transcriptomics analysis, which can provide evidence for the identification of peptides and the protein mass spectrum from MS sequencing. Additionally, the comparative analyses can verify the special biological functions between the venom glands and other tissues (such as muscles and alimentary canals), and thus we can learn more about the process of venom synthesis.

**Figure 3:**
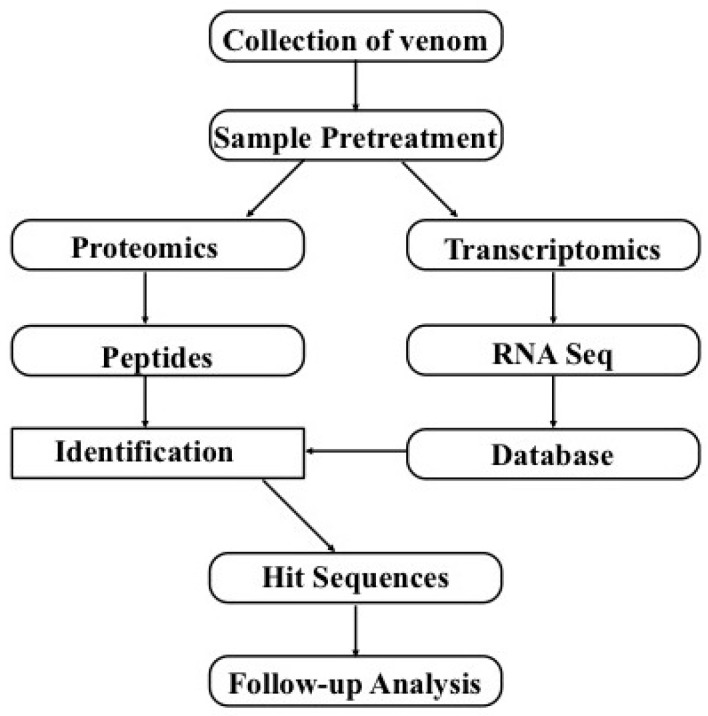
A general strategy for the combination of transcriptomics and proteomics to identify toxin genes on a large scale.

The integration of transcriptomic and proteomic/peptidomic approaches (Figure 3) using bioinformatics can reveal “deep venomics” [9], which can be used to widely explore the toxins present in venoms. Analyzing the toxin sequences with NGS cannot rely only on the basic sequence similarities, since toxins can display high diversities. Meanwhile, skin or other tissues are always included when extracting the venom glands due to their special connection (e.g., fish venom glands are always embedded in the skins), and therefore toxin-like proteins (TLP) in other tissues will influence our annotation results. Hence, proteomics will provide necessary evidence to verify these transcripts. Now this combination method gives access to nearly complete toxin repertoires of all single venoms, because transcript-based databases are illustrative for the certification of peptides and protein expression profiles. Another advance of the combination of transcriptomics and proteomics is to provide insights into the mechanisms of the diversities of toxin peptides at both the cDNA level and post-translational modification (PTM) level.

Our recent combination of transcriptomics and proteomics analyses for the Chinese Yellow catfish [7] and the Chinese tubular cone snail [51] indicated that (i) different mature toxin sequences can originate from one single toxin precursor by alternative splicing, insertion, premature transcription termination, or PTMs [7,51]; (ii) large discrepancies between proteome and transcriptome data were shown in the venom gland of Chinese yellow catfish [7]. This phenomenon was also reported in the central American snake [2]. Interestingly, we found that sometimes toxins that are predicted from transcriptome data cannot be supported by the proteome data. Conversely, some toxins in the proteome have no corresponding transcripts. The source of these discrepancies may be due to the selective expression of venom peptides or proteins from the genes [25]. Sometimes, venom samples for NGS are extracted from one group of samples while proteomic material is only collected from the other group, because the quantity of venom in one venom gland is always not sufficient. There are still some unknown types of PTMs, which may also provide reasonable explanations [25]. An alternative theory was proposed in a recent study on the origin of the ontogenic shift in the venom content of the Central American rattlesnake [2]: that miRNA levels are the main factor that modulates venom composition as the relative toxin transcriptional activity was similar at all the development stages.

## 6. Summary

Venomous marine animals have been revealed to be an important resource for pharmacological tools with promising biological activities. These compounds not only have novel chemical structures but also new functions and/or functional mechanisms. In order to conduct preclinical and clinical trials and further develop a promising lead into a marketed drug, a sustainable supply of these toxins is necessary and challenging. For venomous fish, we are glad to launch the Fish T1K program [62] with a project on the *Comparative Genomics of Fish Venoms*, which will greatly enrich our marine toxin databases so as to overcome the obstacles from lacking reference sequences. Improvements in technologies, such as sampling strategies, nanoscale nuclear magnetic resonance (NMR) for structure determination, full-length chemical synthesis, data opening and exchange, and collaborations between research groups, are all crucial for the successful development of marine toxins as drug leads. However, a high degree of innovation in the field of marine toxins will generate a new wave of new drug research and development in the coming future. Interdisciplinary research using new technologies will be essential for the future success of marine toxins as new therapeutic chemical entities that can make significant contributions to the cure of human diseases. Through the combination of transcriptomics and proteomics, the contribution of marine toxins to the future pharmaceuticals seems to be more promising.

## Figures and Tables

**Table 1:** Summary of sequence number in our achieved toxin database (updated in January 2017).

Group of Species	Taxonomy Name	Numbers of Sequences
Snakes	Serpents	1684
Scorpions	Scorpions	1510
Spiders	Araneae	1391
Cone snails	Conus	3860
Sea anemones	Actiniaria	308
Insects	Hexapoda	162
Fish	Teleostei	44
Mammals	Mammalias	106
Lizards	Heloderma	241
Jellyfish	Cubomedusae/Scyphozoa	175
Sea stars	Asteroidea	8
Hydra	Hydroida	14
Worms	Cerebratulus	5
Forg, Toad	Amphibia	85
Sea-urchin	Echinoidea	2
Sea hare	Aplysiomorpha	44
Scolopendra	Myriapoda	49
All	Metazoa	9688
